# Supplementary material for: Molecular mechanism of mitochondrial phosphatidate transfer by Ups1
Source: Commun Biol. 2020 Aug 25;3:468. doi: 10.1038/s42003-020-01121-x (PMC7447767; doi:10.1038/s42003-020-01121-x)
Supplement: Supplementary file 1 — Supplementary Information [file 42003_2020_1121_MOESM1_ESM.pdf]

Supplementary Figures

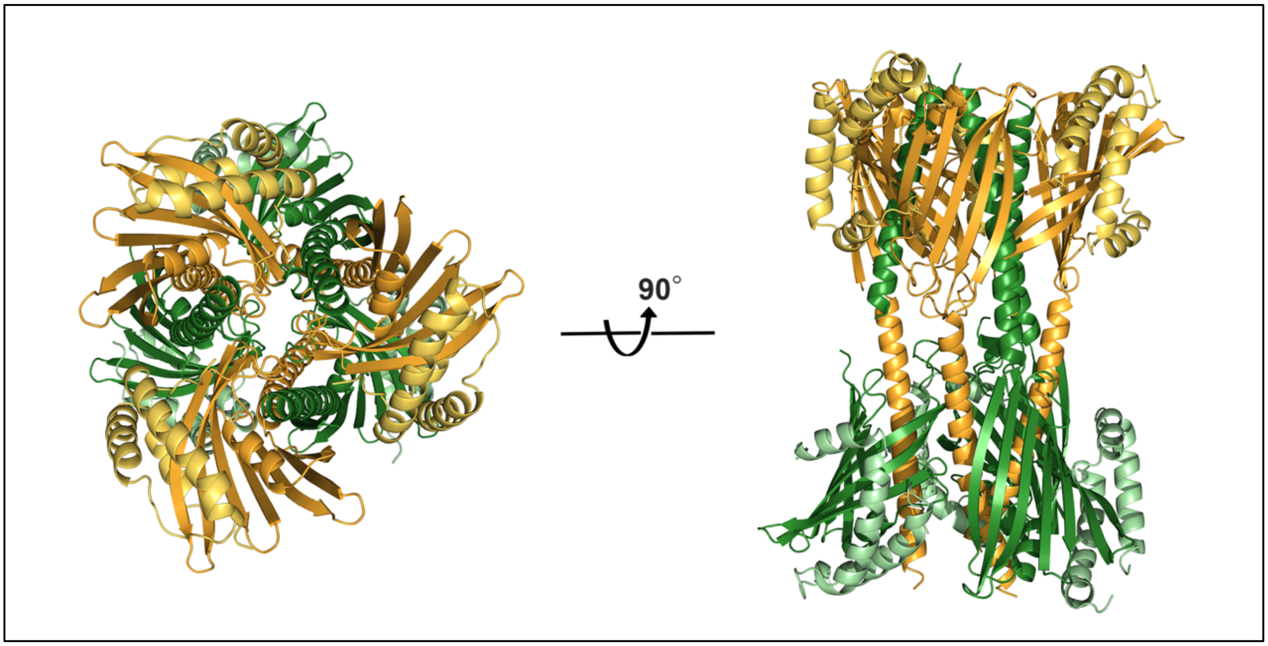

Supplementary Figure 1 | The asymmetric unit in our Ups1/Mdm35 crystal structure (PDB code 5JQL) contains six Ups1/Mdm35 heterodimers.

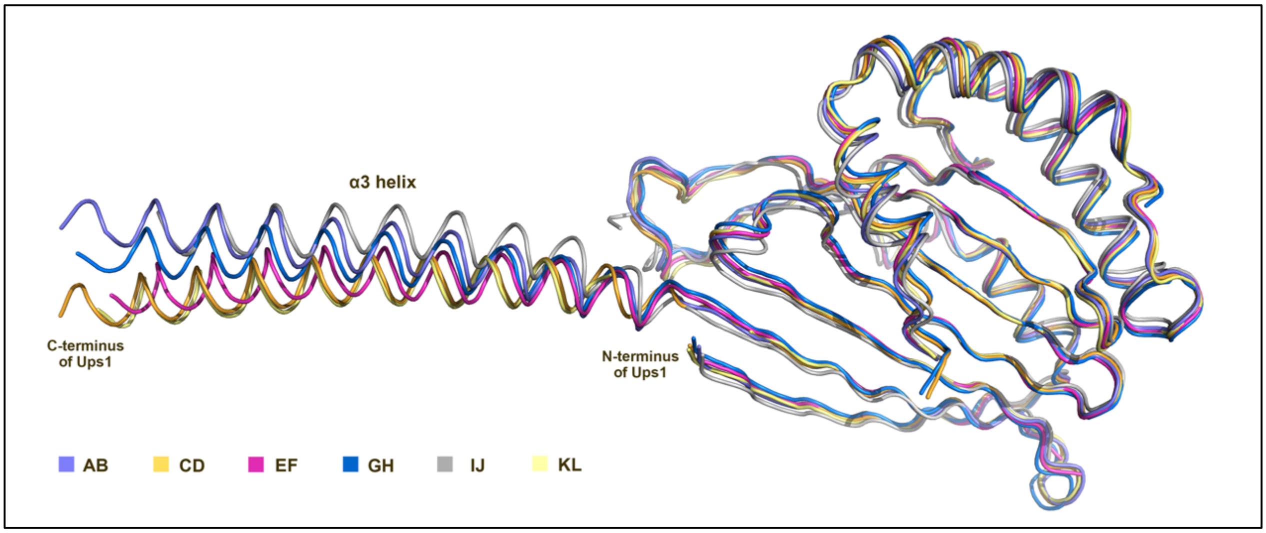

Supplementary Figure 2 | Superposition of six Ups1/Mdm35 molecules in an asymmetric unit of our Ups1/Mdm35 crystal structure (PDB code 5JQL). AB, CD, EF, GH, IJ and KL represent six Ups1/Mdm35 molecules, respectively.

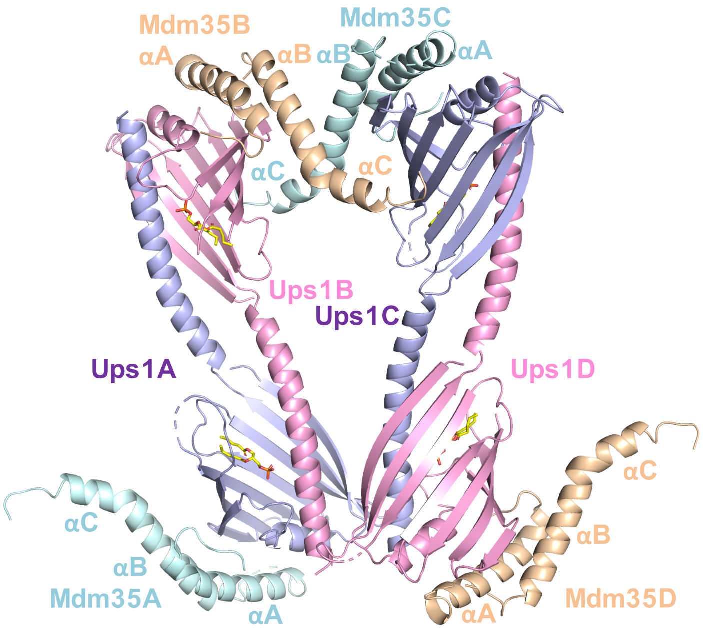

Supplementary Figure 3 | Mdm35 molecules in the structure of Ups1/Mdm35-DHPA are domain swapped. The αC-helices (residues 62–74) of Mdm35B and Mdm35C are swapped, so the αC-helix of Mdm35C and Ups1B, or the αC-helix of Mdm35B and Ups1C interact.

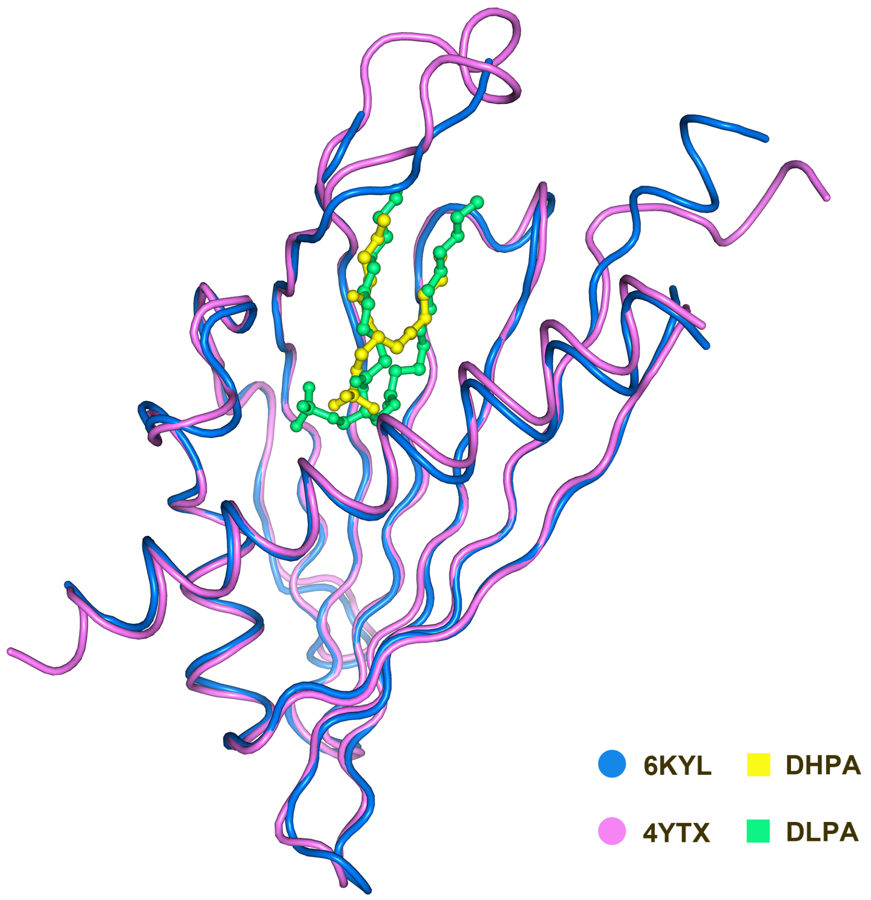

Supplementary Figure 4 | Structural superposition of Ups1/Mdm35-DHPA (PDB code 6KYL) and Ups1/Mdm35-DLPA (PDB code 4YTX).

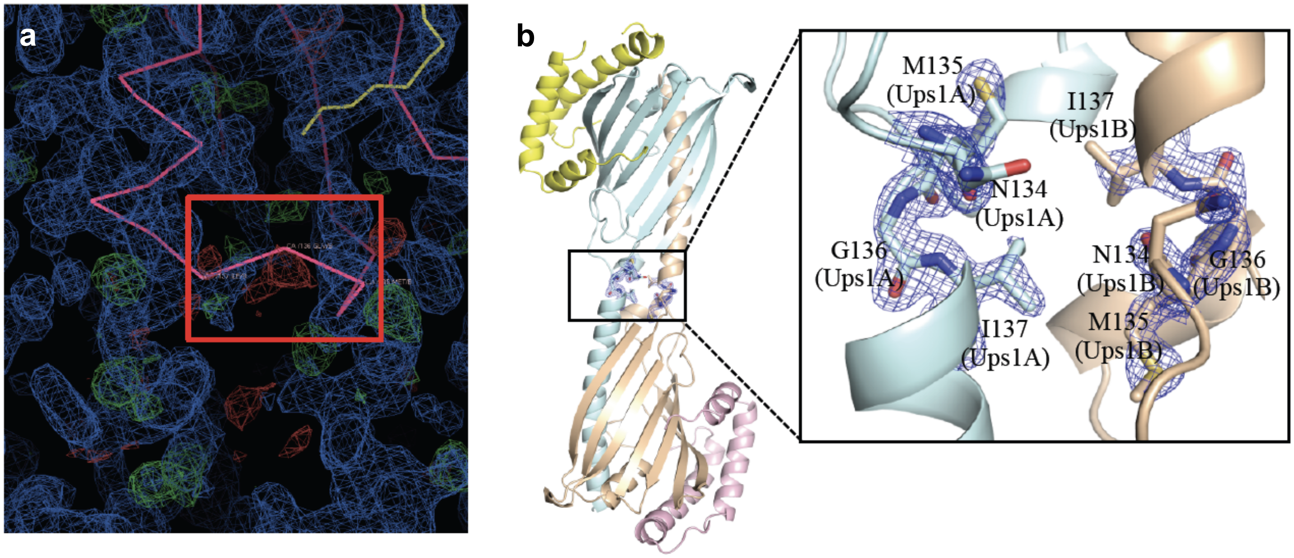

Supplementary Figure 5 | Structural rebuilding of Ups1/Mdm35-DPPA (PDB code 4XIZ). **a**. Structure of Ups1/Mdm35-DPPA is shown as Cα plus ligands before rebuilding. Ups1/Mdm35 is colored in pink; DPPA is colored in yellow. Red panel shows the loop (N134-I137) between the α2 helix and β7 of Ups1. **b.** Structure of Ups1/Mdm35-DPPA after rebuilding. Ups1 molecules are colored in light blue and wheat. Mdm35 molecules are colored in yellow and pink. *Fo-Fc* omit map of the loop (N134-I137) between α2 helix and β7 of Ups1 is contoured at 2σ and colored in blue.

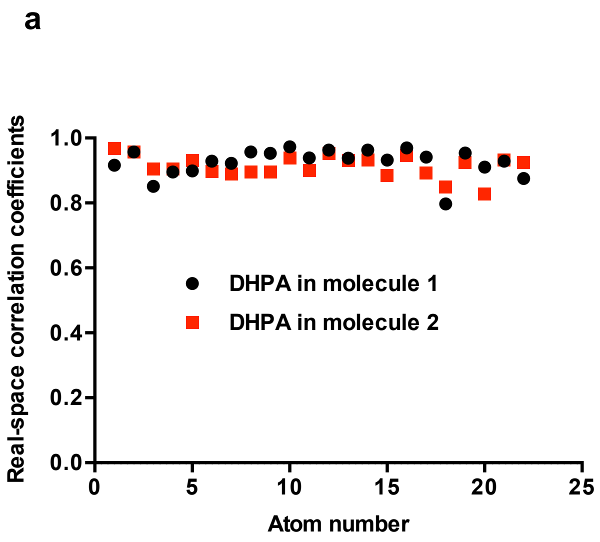

b

Atom No.	Name	Atom No.	Name
1	O1	12	C5
2	O2	13	C6
3	P1	14	C7
4	O3	15	C8
5	O4	16	O7
6	C1	17	C9
7	C2	18	O8
8	C3	19	C10
9	O5	20	C11
10	C4	21	C12
11	O6	22	C13

Supplementary Figure 6 | Validation of DHPA in the structure of Ups1/Mdm35-DHPA.

a. The real-space correlation coefficient (RSCC) of the ligand DHPA against the electron density map is plotted vs. the atom number. The RSCC was computed using PHENIX. **b.** The detailed atom information for every atom number is shown in (**a**).

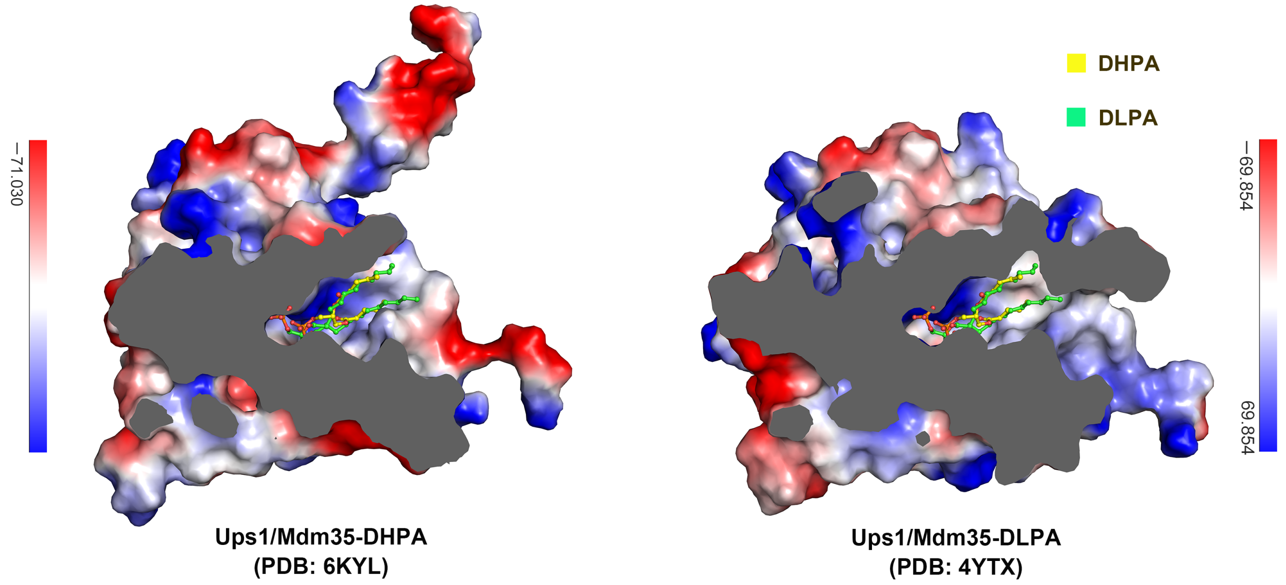

Supplementary Figure 7 | Cutaway representation of the Ups1/Mdm35-DHPA and Ups1/Mdm35-DLPA at the level of the PA binding pocket, and colored according to the surface electrostatic potential. The two structures had been superimposed, but are shown here separately. To better compare the positions of DHPA and DLPA, these two lipid molecules are superimposed in each structure and shown as stick models. DHPA and DLPA are shown yellow and green， respectively. And oxygen atoms are colored red and phosphorus atoms are colored orange.

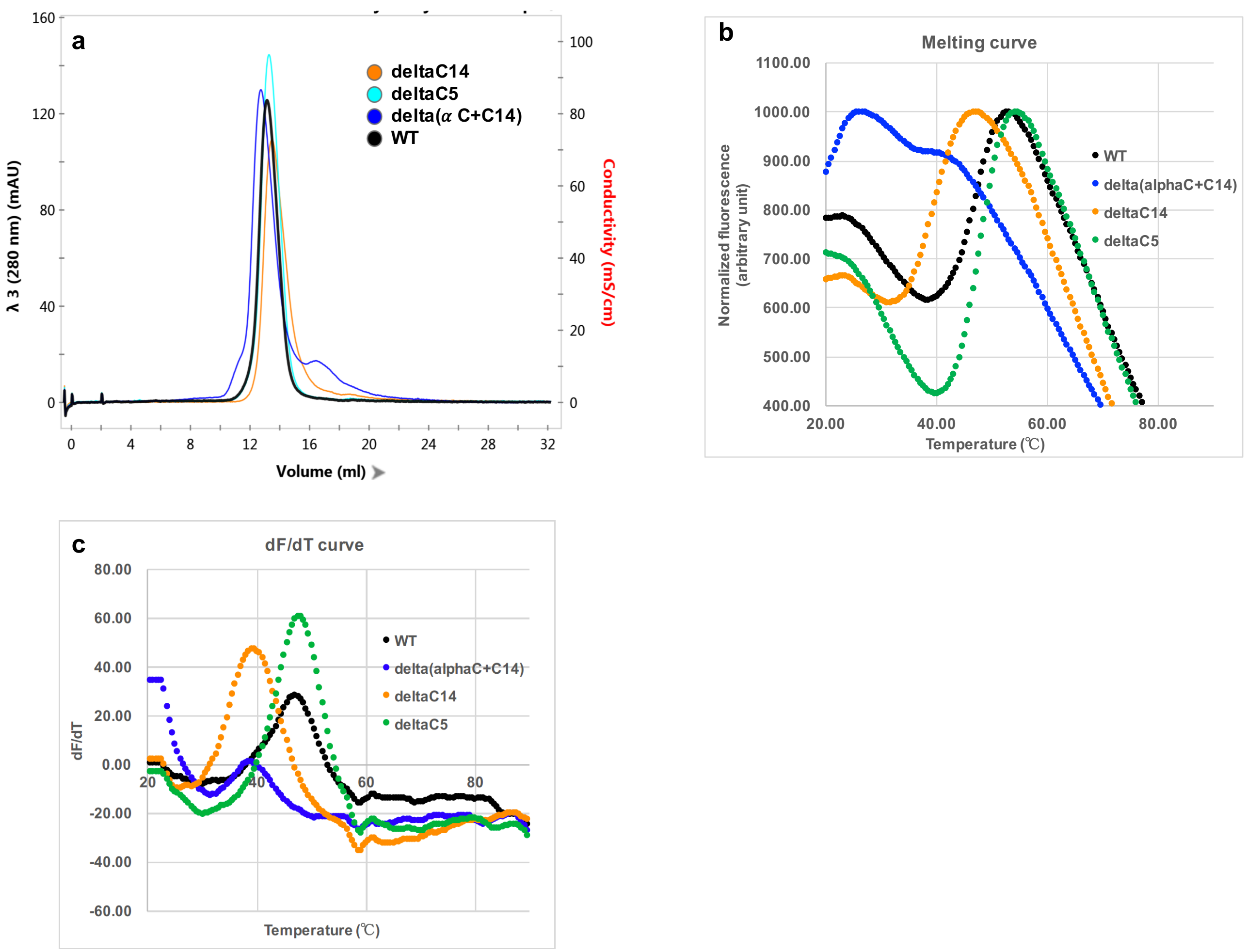

Supplementary Figure 8 | Analyses of △C5, △C14 and △(αC+C14) mutants of Ups1/Mdm35. a. Gel filtration profiles of the WT Ups1/Mdm35 and its mutants**. b** and **c.** Thermal shift assay of the WT Ups1/Mdm35 and its mutants, showing the original melting curves (**b**) and the corresponding first derivative curves (**c**). Compared with the WT, the melting temperatures of △C14 and △(αC+C14) mutants are lower, which indicates a reduced interaction between Ups1 and Mdm35.

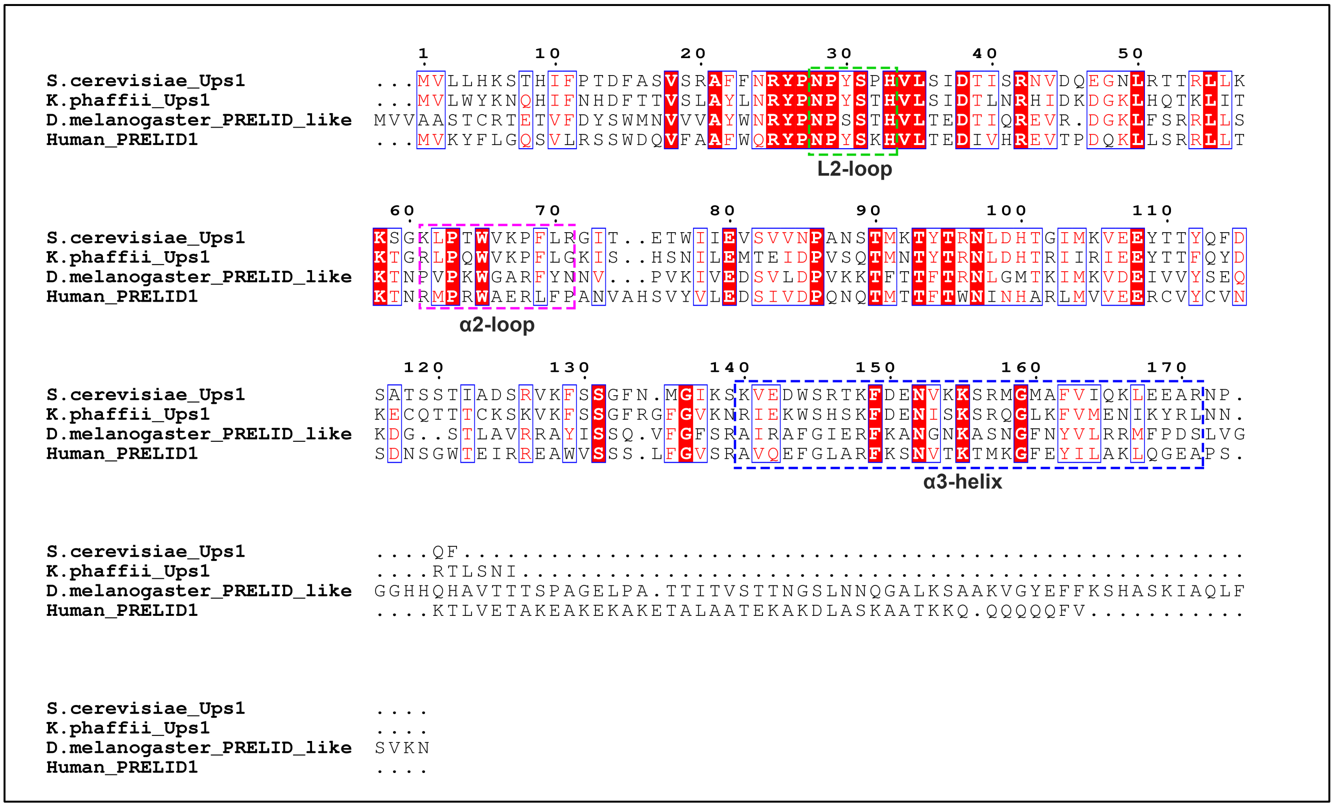

Supplementary Figure 9 | Sequence alignment of Ups1 homologues. Invariant residues are shaded in red, conserved residues are colored in red and framed in blue box. The three membrane-binding elements of Ups1, including the L2-loop, α2-loop, and α3-helix, are framed by dashed lines.

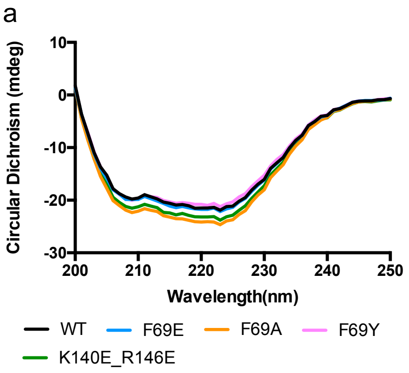

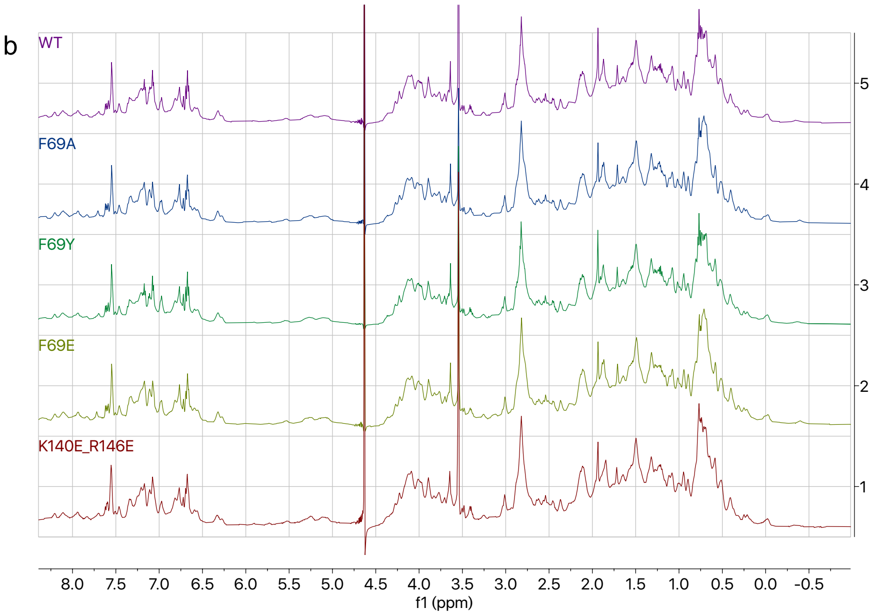

Supplementary Figure 10 | Analyses of the WT Ups1/Mdm35 and its mutants. a. CD spectra of the WT Ups1/Mdm35 and the mutants as indicated. **b**. One dimensional (1D) ^{1}H NMR spectra of the WT Ups1/Mdm35 and the mutants as indicated. The profiles of the WT and the mutants are substantially similar, suggesting they share a very similar conformation.

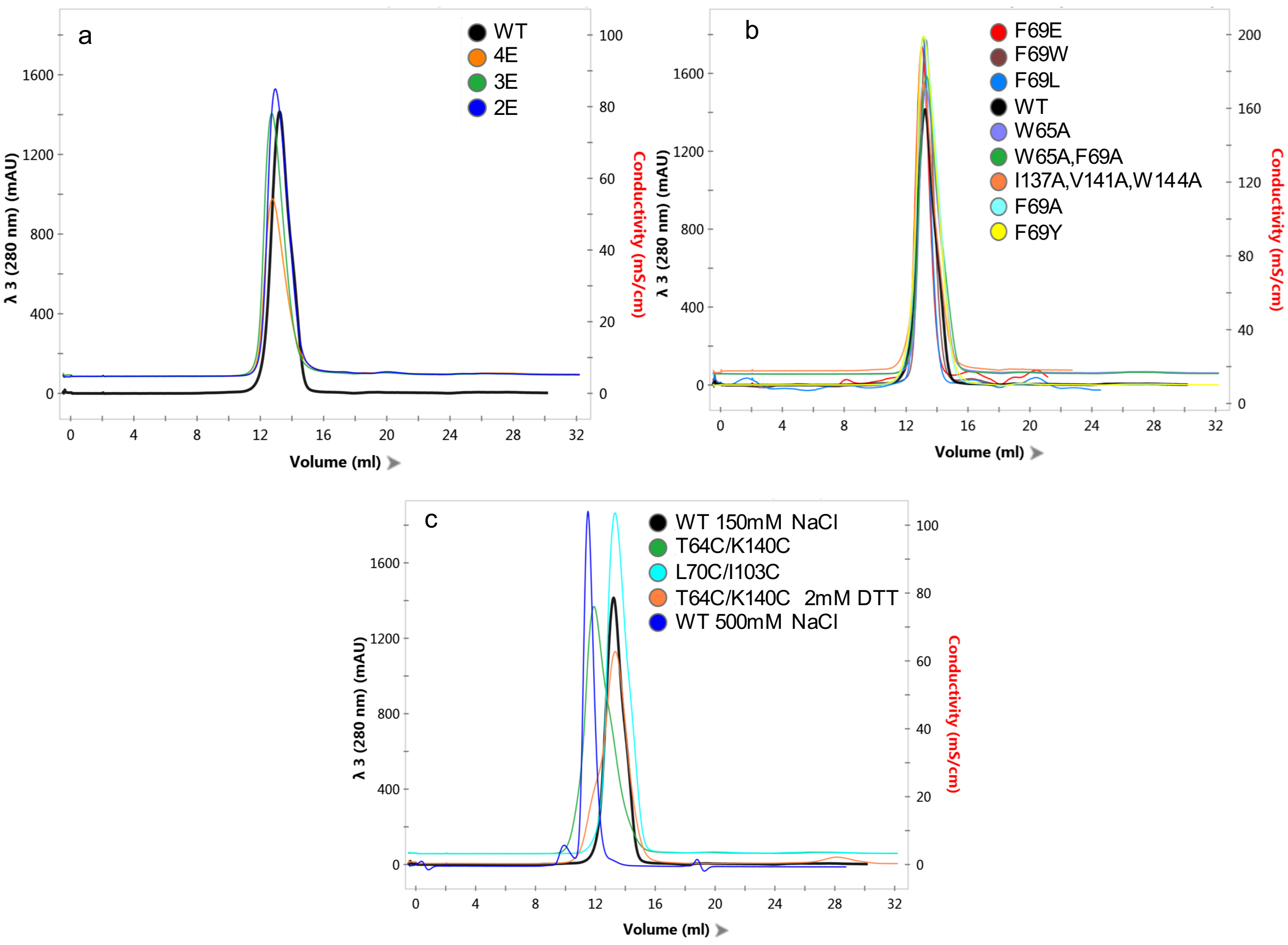

Supplementary Figure 11 | Gel filtration profiles of the WT Ups1/Mdm35 and its mutants. Under 150mM NaCl condition, Ups1/Mdm35 exists as a monomer, while under 500mM NaCl condition, Ups1/Mdm35 seems to exist as a dimer. The elution profiles of all mutants except the T64C/K140C mutant are very similar to that of the WT under 150mM NaCl condition. By adding DTT to the solution at a final concentration of 2 mM, the elution profile of the T64C/K140C mutant becomes similar to that of the WT. Without DTT, the elution peak of the T64C/K140C mutant is between dimer and monomer elution positions of Ups1/Mdm35. This result is consistent with the non-reducing SDS-PAGE behavior of this mutant (**Fig. 4g**).

Disulfied bond analysis of T64C/K140C mutant

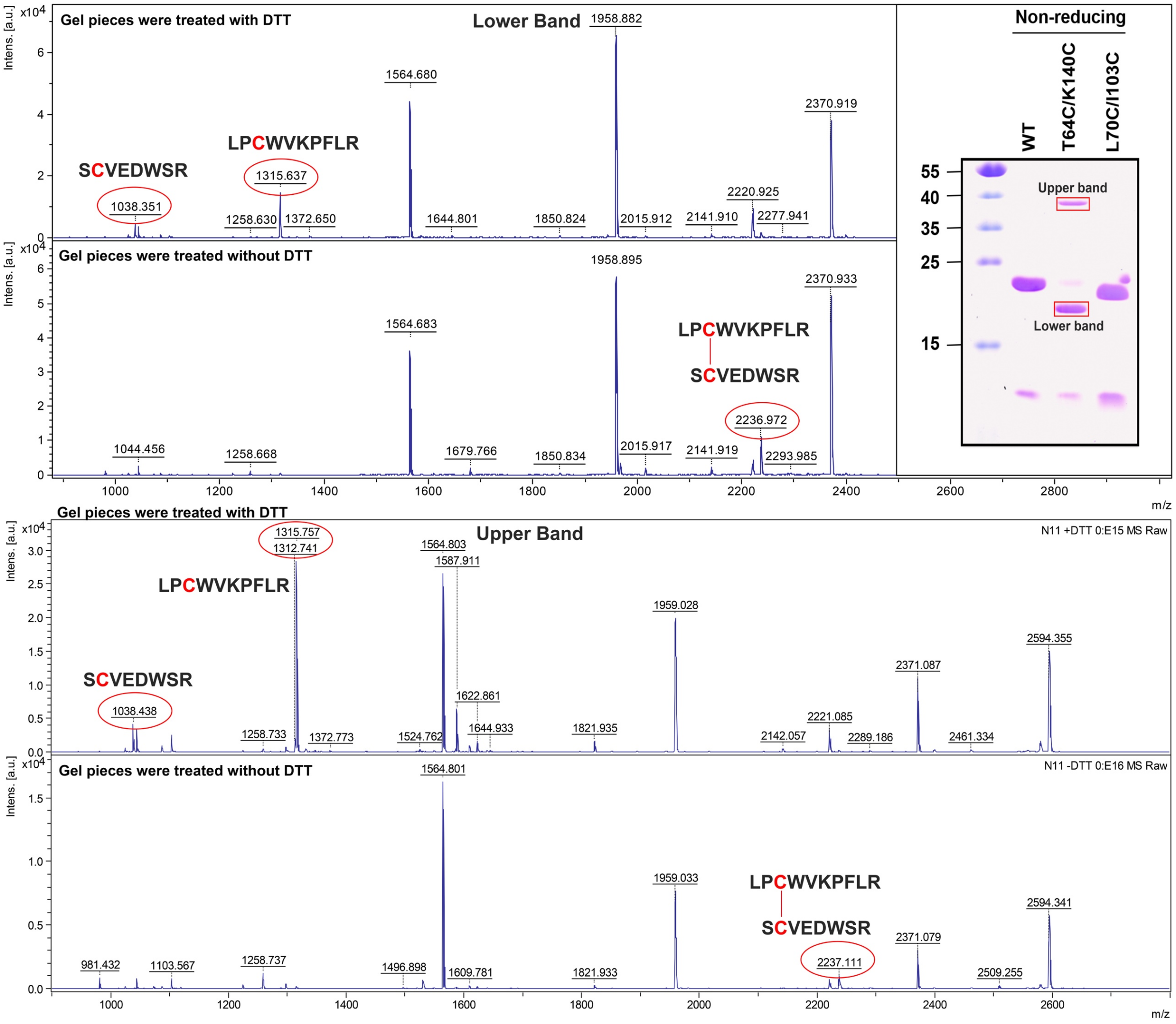

Disulfied bond analysis of L70C/I103C mutant

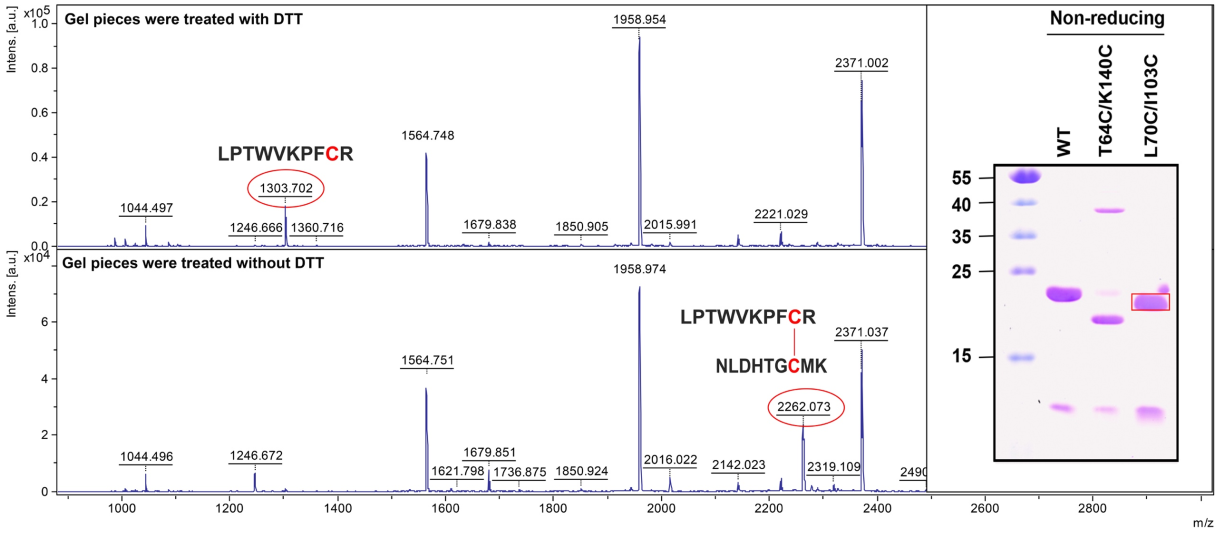

Supplementary Figure 12 | Mass spectrometry analyses of the disulfide bonds in T64C/K140C and L70C/I103C mutants. The MALDI-TOF (matrix assisted laser desorption ionization time of fly) mass spectra of the target bands (framed in red boxes) are presented with the SDS-PAGE gel shown at right. The gel sample from the target bands were treated with or without DTT before MALDI-MS analysis. For the T64C/K140C mutant, both the upper band (the band marked with a black star in **Fig. 4g**) and the lower band were analyzed.

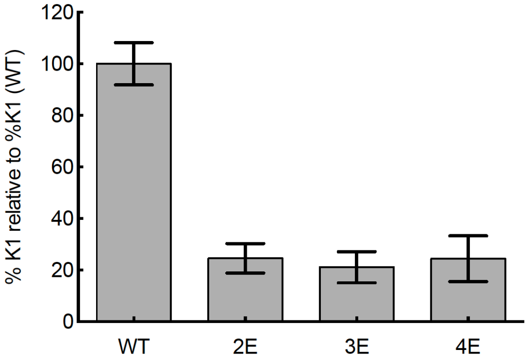

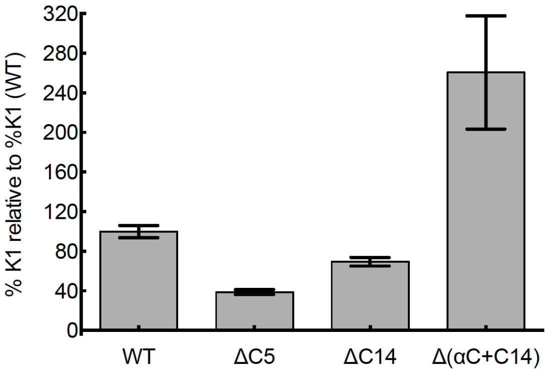

Supplementary Figure 13 | K1 values of the WT of Ups1/Mdm35 and the indicated mutants (see Figs. 2 and 3). K1 is calculated according to the equation (18) of the **Supplementary Text**.

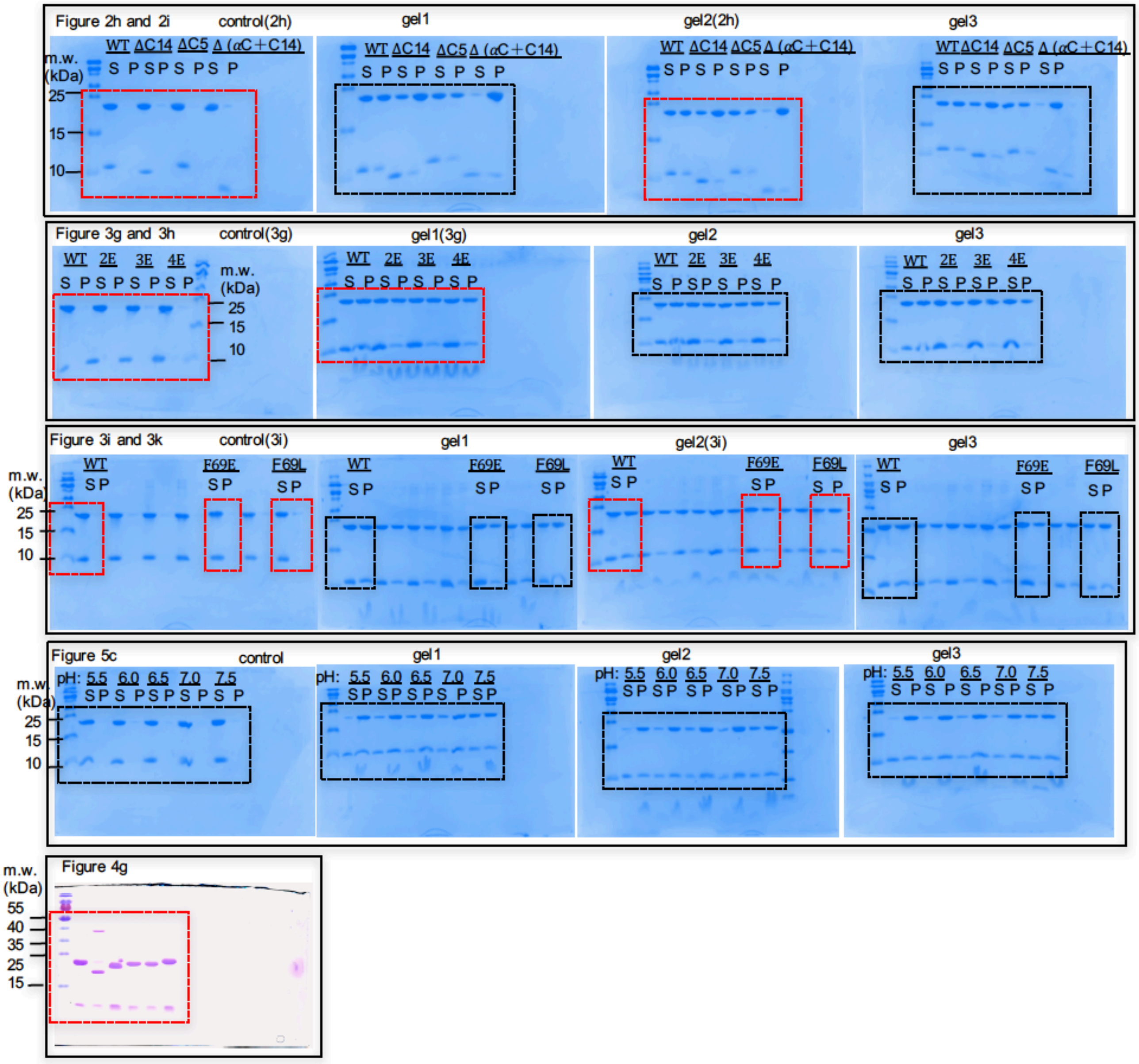

Supplementary Figure 14 | Uncropped raw gel images for panels in Figs. 2, 3, 4 and 5. Red broken boxes mark the borders of the final cropped images, which were used for both corresponding presentation and data analysis. Black broken boxes mark the borders of the gels that are used for data analysis.

Supplementary Tables

Supplementary Table 1. Comparison of the distances between the α2-loop and N-α3-helix of Ups1 in different states

State	Source	P63-V141	P63-W144	W65-V141	W65-W144	Interaction area*
Membrane-anchored state	Simulated structure of $Ups1_{free}$ at 1023ns	8.5	7.2	6.5	8.3	499
Apo-State	5JQM (chain B)	8.6	7.9	8.8	10.5	350
	4YTW(chain D)	10.2	8.2	11.3	11.2	234
PA-bound State	4YTX (chain B, DLPA)	14.4	12.9	15.3	15.1	0
	6KYL (chain D, DHPA)	15.1	14.3	16.8	18.1	0
X-State	4YTX (chain L, ?)	20.3	19.2	20.9	21.0	0

The unit of distance is Å. The unit of interaction area is $Å^2$. * The interaction area is between the α2-loop (K61-I73) and the N-α3-helix (G136-T147) of Ups1.

Supplementary Table 2. The number of net charges carried by Ups1 under different pH

	pH5.5	pH6.0	pH6.5	pH7.0	pH7.5
5JQM (Apo state)	12	8	8	8	8
4YTW (Apo state)	11	9	9	8	7
Ups1free_1023ns (membrane-anchored state)	11	10	8	8	8
4YTX (X-state)	10	9	7	7	7
4YTX (DLPA-bound state)	9	8	7	6	6
Only Ups1 parts of these structure were used for calculation. The net charge was calculated by PDB2PQR Server..					

Supplementary Table 3. Primers used in molecular cloning of Ups1/Mdm35 and Ups1-Mdm35 fusion

**pETDuet-1-Ups1/Mdm35 construct**	
Ups1_F	5’- CGGGATCCGATGGTCCTTTTACACAAAAGC-3’
Ups1_R	5’- ACGCGTCGACTCAAAACTGAGGATTTCTCGC-3’
Mdm35_F	5’-GGAATTCATATGGGGAATATAATGTCAGCT-3’
Mdm35_R	5’-CCGCTCGAGTCATTTGTCAACTTCTTTTAG-3’
**pETDuet-1-Ups1-Mdm35 fusion (UMF) construct**	
UMF_Ups1_F	5’-CGGGATCCGATGGTCCTTTTACACAAAAGC-3’
UMF_Ups1_R	5’-CGGCCCCTGGAACAGAACTTCCAGAAACTGAGGATTTCTCGCCTC-3’
UMF_Mdm35_F	5’-CTGGAAGTTCTGTTCCAGGGGCCGATGGGGAATATAATGTCAGCT-3’
UMF_Mdm35_R	5’-ACGCGTCGACTCATTT GTCAACTTCTTTTAG-3’

Supplementary Discussion

Molecular model for Ups1/Mdm35-mediated PA transport and kinetic analysis

The molecular model for Ups1/Mdm35-mediated PA transport is shown as **Fig. 6** and **Supplementary Movie 4**. It is described in details here.

First, when Ups1/Mdm35 approaches lipid bilayers, Ups1/Mdm35 interacts with the membrane through the membrane-binding residues of Ups1, including N28–H33 of the L2-loop, and the hydrophobic and positively charged residues of the α2-loop and the C-terminal long α3-helix. Although these membrane-binding residues are highly flexible in the presence of Mdm35, they can still interact with the membrane and facilitate membrane-binding of Ups1/Mdm35 as an integral part (membrane-bound Ups1/Mdm35).

Second, Mdm35 dissociates from Ups1, initiating through αC-helix detachment, followed by detachment of αA and αB helices, liberating Ups1 residues at the Ups1/Mdm35 interface and the resulting allosteric effects alleviate the flexibility of the membrane-binding residues of Ups1. Under this circumstance, hydrophobic interactions (mediated by P63, W65, I137, V141, and W144) between the α2-loop and N-α3-helix are enhanced, resulting in F69 membrane insertion. This event makes the COM of Ups1 further approach the lipid bilayer (membrane-anchored Ups1), which is important for PA extraction.

Third, the negatively charged phosphate head of PA is attracted to the positively charged residues H33, K61, K148, and K155 and interacts with them, inducing conformational changes in the N-α3-helix and α2-loop. They migrate in opposite directions to enlarge the entrance of the PA-binding pocket, allowing the two long acyl tails of PA to enter the pocket. W65 is inserted into the membrane as an F69 substitute. When PA completely enters the binding pocket, the N-α3-helix swings slightly inward to prevent PA exit from the pocket.

Fourth, Mdm35 interacts again with PA-bound Ups1 to form Ups1/Mdm35-PA. In the fifth

and final step, Ups1/Mdm35-PA is released from the membrane to the aqueous environment. PA release by Ups1/Mdm35 is simply a reverse process of PA extraction and is not further discussed here.

On the basis of our model and the previously reported kinetic model for phospholipid binding and transfer[1], the kinetics of Ups1/Mdm35-mediated PA transfer can be described as follows:

$$
\begin{aligned}
&U/M + D \underset{k_{-1}}{\overset{k_1}{\rightleftarrows}} U/M \bullet D \underset{k_{-2}}{\overset{k_2}{\rightleftarrows}} U \bullet D + M \\
&U \bullet D \underset{k_d}{\overset{k_a}{\rightleftarrows}} U - PA \bullet D \\
&U - PA \bullet D + M \underset{k_{-3}}{\overset{k_3}{\rightleftarrows}} U - PA/M \bullet D \underset{k_{-4}}{\overset{k_4}{\rightleftarrows}} U - PA/M + D
\end{aligned}
\quad , \qquad \text{(a)}
$$

$$
\begin{aligned}
&U - PA/M + A \underset{k_5}{\overset{k_{-5}}{\rightleftarrows}} U - PA/M \bullet A \underset{k_6}{\overset{k_{-6}}{\rightleftarrows}} U - PA \bullet A + M \\
&U - PA \bullet A \underset{k_{a'}}{\overset{k_{d'}}{\rightleftarrows}} U \bullet A \\
&U \bullet A + M \underset{k_7}{\overset{k_{-7}}{\rightleftarrows}} U/M \bullet A \underset{k_8}{\overset{k_{-8}}{\rightleftarrows}} U/M + A
\end{aligned}
\quad , \qquad \text{(b)}
$$

where U, M, U/M, D, A, and PA represent Ups1, Mdm35, Ups1/Mdm35, donor membrane, acceptor membrane, and PA, respectively; U-PA and U-PA/M PA-bound Ups1 and PA-bound Ups1/Mdm35, respectively; and U/M·D, U/M·A, U-PA/M·D, and U-PA/M·A the corresponding membrane-bound complexes. The reaction constants for each step are described as k_1 to k_8, and k_{-1} to k_{-8}. The association/dissociation constants of PA to Ups1 are denoted as k_a, $k_{a'}$, k_d, and $k_{d'}$. Reaction (a) represents the binding of Ups1/Mdm35 to the donor membrane and subsequent PA extraction, whereas reaction (b) represents the binding of Ups1/Mdm35 to the acceptor membrane and subsequent PA release. Reaction (b) is the reverse of reaction (a). However, because of the different composition of the donor and acceptor membrane, the

reaction constants differed in these two reactions.

In liposome co-sedimentation assay, donor and acceptor membrane are identical, only reaction (a) needs to be considered. When the reactions (a) reach equilibrium, six equations can be derived as follows,

$$\frac{d[U/M]}{dt} = k_{-1}[U/M \bullet D] - k_1[U/M][D] = 0 \tag{1}$$

$$\frac{d[U/M \bullet D]}{dt} = k_1[U/M][D] - k_{-1}[U/M \bullet D] + k_{-2}[U \bullet D][M] - k_2[U/M \bullet D] = 0 \tag{2}$$

$$\frac{d[U \bullet D]}{dt} = k_2[U/M \bullet D] - k_{-2}[U \bullet D][M] + k_d[U-PA \bullet D] - k_a[U \bullet D] = 0 \tag{3}$$

$$\frac{d[U-PA \bullet D]}{dt} = k_a[U \bullet D] - k_d[U-PA \bullet D] + k_{-3}[U-PA/M \bullet D] - k_3[U-PA \bullet D][M] = 0 \tag{4}$$

$$\frac{d[U-PA/M \bullet D]}{dt} = k_{-4}[U-PA/M][D] - k_4[U-PA/M \bullet D] + k_3[U-PA \bullet D][M] - k_{-3}[U-PA/M \bullet D] = 0 \tag{5}$$

$$\frac{d[U-PA/M]}{dt} = k_4[U-PA/M \bullet D] - k_{-4}[U-PA/M][D] = 0 \tag{6}$$

From (6) we have,

$$k_{-4}[U-PA/M][D] = k_4[U-PA/M \bullet D] \tag{7}$$

We assume that the association/dissociation reactions can be described with similar rate constants for both U/M or U-PA/M (i.e., k4/k-4 is approximately equal to k-1/k1). Then, (7) can be rewritten into:

$$k_1[U-PA/M][D] = k_{-1}[U-PA/M \bullet D] \tag{8}$$

Addition of (1) and (8) yields,

$$K1 = \frac{k_1}{k_{-1}} = \frac{[U/M \bullet D] + [U - PA/M \bullet D]}{\{[U/M] + [U - PA/M]\}[D]} \quad (9)$$

In liposome co-sedimentation assay, we have,

$$[Mdm35]_{total} = [M] + [U/M] + [U/M \bullet D] + [U - PA/M \bullet D] + [U - PA/M] \quad (10)$$

$$[Ups1]_{total} = [U/M] + [U/M \bullet D] + [U \bullet D] + [U - PA \bullet D] + [U - PA/M \bullet D] + [U - PA/M] \quad (11)$$

$$[Mdm35]_{total} = [Ups1]_{total} \quad (12)$$

Based on the scheme of **Fig. 2g**, the following equations can be deduced:

$$[U_C]+[U_A]+[U_B] = [Ups1]_{total} = [M_C]+[M_F]+[M_A] = [Mdm35]_{total} = [Ups1\text{-}Mdm35]_{input} \quad (13)$$

$$[U_C] = [U/M] + [U\text{-}PA/M] \quad (14)$$

$$[U_A]+[U_B] = [U/M \bullet D] + [U \bullet D] + [U\text{-}PA \bullet D] + [U\text{-}PA/M \bullet D] \quad (15)$$

$$[M_C]+[M_F] = [M] + [U/M] + [U\text{-}PA/M] \quad (16)$$

$$[M_A] = [U/M \bullet D] + [U\text{-}PA/M \bullet D] \quad (17)$$

Substitution of (14) and (17) into (9), the equilibrium constant (K1) of Ups1/Mdm35 binding to the membrane can be derived as follows,

$$K1 = \frac{k_1}{k_{-1}} = \frac{[U/M \bullet D] + [U - PA/M \bullet D]}{\{[U/M] + [U - PA/M]\}[D]} = \frac{[M_A]}{[U_C][D]} \quad (18)$$

where $[M_A]$ represents the concentration of Mdm35 bound to Ups1 on the membrane, $[U_C]$ the concentration of Ups1 in solution, and [D] the concentration of the donor membrane (**Fig. 2g**).

We then calculated K1 by using experimental data from liposome co-sedimentation assays (**Supplementary Fig. 13**). We found, K1 of 2E, 3E, and 4E mutants were considerably lower than those of the WT complex, indicating that mutating the positively charged residues at the

Ups1-membrane interface to negatively charged residues decreases the membrane-binding ability of Ups1/Mdm35, which is consistent with our prediction, although the apparent total amount of membrane-bound Ups1 was not considerably reduced (**Fig. 3h**). Furthermore, the K1 of the Δ(αC+C14) mutant was considerably higher than that of the WT complex, suggesting that the mutant binds to the membrane more easily, which is also consistent with our prediction.

Reference

1 Gadella, T. W., Jr. & Wirtz, K. W. Phospholipid binding and transfer by the nonspecific lipid-transfer protein (sterol carrier protein 2). A kinetic model. *Eur J Biochem* **220**, 1019-1028 (1994).
