# Supplementary material for: Molecular mechanism of mitochondrial phosphatidate transfer by Ups1
Source: Commun Biol. 2020 Aug 25;3:468. doi: 10.1038/s42003-020-01121-x (PMC7447767; doi:10.1038/s42003-020-01121-x)
Supplement: Supplementary file 9 — Description of Additional Supplementary Files [file 42003_2020_1121_MOESM9_ESM.pdf]

Description of Additional Supplementary Files

File Name: **Supplementary Movie 1**
Description: Molecular dynamics simulation of Ups1free on the lipid bilayer. Secondary structural elements are shown in cartoon. Alpha helices, beta strands and coils are colored in magenta, yellow and cyan, respectively.

File Name: **Supplementary Movie 2**
Description: Molecular dynamics simulation of Ups1/Mdm35 complex on the lipid bilayer. Secondary structural elements are shown in cartoon. Ups1 and Mdm35 are colored in cyan and orange, respectively.

File Name: **Supplementary Movie 3**
Description: Molecular dynamics simulation of Ups1free on the lipid bilayer from 800 ns to 1023 ns. A front view of the membrane binding interface of Ups1 is presented here. Ups1 is colored in pink. The PA molecule is shown in stick and colored in cyan. Residues in contact with PA during the simulation are shown in stick. Nonpolar residues, polar residues, negatively charged, positively charged, polar and nonpolar residues are colored in red, blue, green and white, respectively.

File Name: **Supplementary Movie 4**
Description: A proposed model for Ups1/Mdm35-mediated PA transport. Apo-Ups1/Mdm35 can adopt at least two conformations (4YTW and 5JQM) in solution and switch freely between them. In 5JQM, the interaction between W65 and the hydrophobic patch composed of I137, V141 and W144, is stronger than in 4YTW. When Apo-Ups1/Mdm35 approaches the lipid bilayer, Ups1/Mdm35 intends to interact with the membrane through the membrane-binding residues of Ups1 as a whole. Then a thermodynamic perturbation of the system will induce the dissociation of Mdm35 from Ups1, which begins with the αC-helix detachment. The resulting allosteric effects alleviate the flexibility of the membranebinding residues of Ups1 and leads to the insertion of F69 into the membrane (MD simulated Ups1, 350ns). At 1023ns, the head of DLPA interacts with Ups1 (MD simulated Ups1, 1023ns). Then DLPA is trying to enter the PA-binding pocket of Ups1 (X-state Ups1, 4YTXKJ). Finally, DLPA enters the pocket completely (DLPA-bound Ups1, 4YTX-AB).

File Name: **Supplementary Data 1**
Description: The raw data of liposome co-sedimentation experiments and the subsequent process of data analysis. The processed data are used to generate statistics in Figs. 2i, 3h, 3j, and 5c.

File Name: **Supplementary Data 2**
Description: The raw data of PA transfer assays and the subsequent process of data analysis. The processed data are used to generate statistics in Figs. 2f, 3f, 3k, 4e, 4h, and 5a.
