# Supplementary material for: Molecular mechanism of mitochondrial phosphatidate transfer by Ups1
Source: Commun Biol. 2020 Aug 25;3:468. doi: 10.1038/s42003-020-01121-x (PMC7447767; doi:10.1038/s42003-020-01121-x)
Supplement: Supplementary file 10 — Reporting Summary [file 42003_2020_1121_MOESM10_ESM.pdf]

nature research

Corresponding author(s): Jun Fan, Fei Sun, Yujia Zhai

Last updated by author(s): Jun 26, 2020

Reporting Summary

Nature Research wishes to improve the reproducibility of the work that we publish. This form provides structure for consistency and transparency in reporting. For further information on Nature Research policies, see Authors & Referees and the Editorial Policy Checklist.

Statistics

For all statistical analyses, confirm that the following items are present in the figure legend, table legend, main text, or Methods section.

n/a	Confirmed	
☐	☒	The exact sample size (*n*) for each experimental group/condition, given as a discrete number and unit of measurement
☐	☒	A statement on whether measurements were taken from distinct samples or whether the same sample was measured repeatedly
☐	☒	The statistical test(s) used AND whether they are one- or two-sided<br>*Only common tests should be described solely by name; describe more complex techniques in the Methods section.*
☒	☐	A description of all covariates tested
☒	☐	A description of any assumptions or corrections, such as tests of normality and adjustment for multiple comparisons
☒	☐	A full description of the statistical parameters including central tendency (e.g. means) or other basic estimates (e.g. regression coefficient) AND variation (e.g. standard deviation) or associated estimates of uncertainty (e.g. confidence intervals)
☐	☒	For null hypothesis testing, the test statistic (e.g. F, t, r) with confidence intervals, effect sizes, degrees of freedom and P value noted<br>*Give P values as exact values whenever suitable.*
☒	☐	For Bayesian analysis, information on the choice of priors and Markov chain Monte Carlo settings
☒	☐	For hierarchical and complex designs, identification of the appropriate level for tests and full reporting of outcomes
☒	☐	Estimates of effect sizes (e.g. Cohen's *d*, Pearson's *r*), indicating how they were calculated

Our web collection on statistics for biologists contains articles on many of the points above.

Software and code

Policy information about availability of computer code

Data collection	Diffraction data were collected at BL17U and BL18U of SSRF (Shanghai Synchrotron Radiation Facility) using GUGI.
Data analysis	HKL2000, SHELXD, SOLVE, RESOLVE, COOT, PHENIX, MolProbity, SPSS 23.0, NAMD 2.11, Pymol

For manuscripts utilizing custom algorithms or software that are central to the research but not yet described in published literature, software must be made available to editors/reviewers. We strongly encourage code deposition in a community repository (e.g. GitHub). See the Nature Research guidelines for submitting code & software for further information.

Data

Policy information about availability of data

All manuscripts must include a data availability statement. This statement should provide the following information, where applicable:

- Accession codes, unique identifiers, or web links for publicly available datasets
- A list of figures that have associated raw data
- A description of any restrictions on data availability

The coordinates and structural factors of crystal structures of selenomethionine-substituted UPS1/Mdm35, the fusion form and DLPA-bound state have been deposited in Protein Data Bank (accession codes: 5JQL, 5JQM, and 6KYL). The data required to generate statistics of sedimentation experiments in Figs. 2i, 3h, 3j, and 5c are provided in Supplementary Data 1. The data required to generate plots of PA transfer assay in Figs. 2f, 3f, 3k, 4e, 4h, and 5a are provided in Supplementary Data 2. Uncropped raw images of gels are shown in Supplementary Figure 14. All other relevant data are available from the corresponding author upon request.

Field-specific reporting

Please select the one below that is the best fit for your research. If you are not sure, read the appropriate sections before making your selection.

☒ Life sciences ☐ Behavioural & social sciences ☐ Ecological, evolutionary & environmental sciences

For a reference copy of the document with all sections, see nature.com/documents/nr-reporting-summary-flat.pdf

Life sciences study design

All studies must disclose on these points even when the disclosure is negative.

Sample size	For liposome co-sedimentation assay and PA transfer activity assay, each measurement was repeated at least three times.
Data exclusions	No data exclusions performed.
Replication	All measurements are reducible.
Randomization	All allocations were randomized.
Blinding	All allocations were blinded.

Reporting for specific materials, systems and methods

We require information from authors about some types of materials, experimental systems and methods used in many studies. Here, indicate whether each material, system or method listed is relevant to your study. If you are not sure if a list item applies to your research, read the appropriate section before selecting a response.

Materials & experimental systems

n/a	Involved in the study	
☒	☐	Antibodies
☒	☐	Eukaryotic cell lines
☒	☐	Palaeontology
☒	☐	Animals and other organisms
☒	☐	Human research participants
☒	☐	Clinical data

Methods

n/a	Involved in the study	
☒	☐	ChIP-seq
☒	☐	Flow cytometry
☒	☐	MRI-based neuroimaging
